# Annotations

**Published:** 1904-11-19

**Authors:** 


### Annotations.

### Juvenile Criminals.

A recent tragic incident reported from New York, in which a youngster of two-and-a-half years deliberately smashed the skull of his baby sister, has, not unnaturally, received some attention both in the medical and lay Press, and it appears that the occurrence is not without parallels. Thus a similar case occurred in France in August last, a girl of two years having deliberately cut the throat of her infant brother. The suggestion in each of these instances is that the murderer was actuated by jealousy aroused by the attention paid to the later addition to the family circle. One writer regards such acts as "due to the family relations of our complex social arrangements," and expresses wonder that "such evil results do not more often accrue." He considers the deed "so pure a reflex to the stimulation that it is difficult to see the necessity for invoking any pathological factor." The meaning of this phrase is not very manifest, but it appears to involve the startling proposition that the killing of young infants by their elder brothers or sisters must be classed as a strictly physiological action. Another writer takes note of these incidents as samples of a growing precocity in crime, which has for some time engaged the attention of "phrenologists" in both Europe and America. In 50 years, we are told, the criminal percentage of the population of France has increased 33 per cent., and the increase is most marked among the young. Similar figures are quoted from the United States; and in Prussia, it is said, between the years 1884 and 1898 nearly 1,000 children committed suicide. All this forms, indeed, a very melancholy picture, and the pessimist may be expected to find in it a convincing illustration of his doleful creed. Less philosophic persons will recall for their comfort the undoubted evidence of a steady decrease of serious crime, and will remember how misleading are facts and figures when detached from their proper setting.

### Chemist-Opticians.

When it was announced some few months ago that a certain number of pharmacists had formed themselves into a "Society of Chemist-Opticians," there seemed no reason to believe that the movement was other than one of a few individuals anxious to enter themselves as rivals to the prescribing spectacle-maker, and to pose before the public as qualified "optologists." However much one might regret the association of the craft of pharmacy with such ambitions, there was no ground for believing that the official leaders of the art would look otherwise than askance at the "chemist-optician." It is true that one member of the Council of the Pharmaceutical Society had described the process of sight-testing as "a most appropriate side-line for a chemist," but this was regarded merely as an individual deliverance by a person of no considerable weight or responsibility. Neither in this nor in any other incident was there ground for suspecting that the Society in its official and corporate capacity would afford continuance to a scheme condemned by the medical profession as adverse to the public interest. Whatever may be true of a number of individual chemists and druggists, official pharmacy at least has kept its skirts free from the contamination of humbug and quackery. But a recent intimation in the *Pharmaceutical Journal* affords some ground for apprehension in regard to the future in this respect. We are there told that "it is hoped" a meeting of the chemist-opticians will shortly be held in the Pharmaceutical Society's House in Bloomsbury Square for the purpose of hearing a lecture on "The Use of the Ophthalmoscope and the Retinoscope in Detecting Diseases of the Eye." If this "hope" is realised the Pharmaceutical Society will incur a very grave responsibility; and, in spite of the attitude of its official journal, we are reluctant to believe that the Council will sanction such a proposal. If the Pharmaceutical Society is to lend official support to the organisation of the chemist-opticians, it will be high time for the medical profession to reconsider its relationship to the Society.

### Seats for Tramway Servants.

Although the Highways Committee of the London County Council recently declared it to be undesirable that seats should be provided for the drivers and conductors on the trams under their jurisdiction, we cannot doubt that their decision will be over-ruled. The London County Council have shown such a laudable desire to treat their servants reasonably that we are convinced they will not permit it to be said that, in the face of the statement of 200 medical men as to the evils of constant standing, they will not make a concession which is demanded alike in the interests of health, and in those of common humanity. Sir William Collins, who has all along been a strong advocate of the concession, may be relied upon not to let the matter drop until the objections of the Highways Committee have been disposed of. It appears to us that these are of a very feeble character. Against the contention that it would be incompatible with the efficient working of the tramways, or the safety of the public, for the drivers to sit down while they are on duty, and that the provision of seats for conductors would conduce to inattention, may be set the fact that in Australia electric tram-cars seats are allowed without any ill results. The reform was not adopted at the Antipodes, however, until public opinion insisted upon it, and unless the London County Council promptly recognise the unanswerable force of expert testimony as to its necessity, public opinion here must be equally insistent. It is now more than 18 months since the Rev. Dr. Darlington, vicar of St. Mark's, Kennington, first took up the cudgels for the men, and pleaded that the supply of a small collapsible or shut-up temporary seat would be a simple and inexpensive mode of meeting the need. We are not, therefore, surprised that the drivers and conductors are disappointed that nothing has yet been done. They do not earn their moderate wages easily. They are often at work continuously for nine or ten hours, and they are frequently away from home from half-past seven in the morning till after midnight. That they should be compelled to stand all the time they are on duty, when it has clearly been proved unnecessary, constitutes a grievance which calls for redress.

